# Supplementary material for: Jamaican fruit bats’ competence for Ebola but not Marburg virus is driven by intrinsic differences
Source: Nat Commun. 2025 Mar 25;16:2884. doi: 10.1038/s41467-025-58305-4 (PMC11937316; doi:10.1038/s41467-025-58305-4)
Supplement: Supplementary file 1 — Supplementary Information [file 41467_2025_58305_MOESM1_ESM.pdf]

Supplementary Materials for

Jamaican fruit bats' competence for Ebola but not Marburg virus is driven by intrinsic differences

Sarah van Tol[1], Julia R. Port[1,2], Robert J. Fischer[1], Shane Gallogly[1], Trent Bushmaker[1], Amanda Griffin[1], Jonathan E. Schulz[1], Aaron Carmody[3], Lara Myers[3], Daniel E. Crowley[4], Caylee A. Falvo[4], Jade C. Riopelle[1], Arthur Wickenhagen[1], Chad Clancy[5], Jamie Lovaglio[5], Carl Shaia[5], Greg Saturday[5], Jessy Prado-Smith[5], Yi He[6], Justin Lack[3], Craig Martens[3], Sarah L. Anzick[3], Lon Kendall[7], Tony Schountz[7], Raina K. Plowright[4], Andrea Marzi[1], Vincent J. Munster[1]

This file includes:

Supplementary Table 1. Hematology of EBOV and MARV virus infected JFBs
Supplementary Table 2. Clinical chemistry of healthy control and EBOV or MARV-infected JFBs
Supplementary Table 3. Differentially Upreglated genes in EBOV or MARV-infected JFBs compared to healthy controls
Supplementary Table 4. Differentially Upreglated genes in EBOV-infected compared to MARV-infected JFBs
Supplementary Table 5. RT-qPCR primers for JFB and ERB immune genes and viruses
Supplementary Figure 1. Challenge with EBOV or MARV does not induce histopathologic changes in JFBs
Supplementary Figure 2. EBOV infection induces mild interferon-stimulated gene expression in the lung
Supplementary Figure 3. BCR sequencing
Supplementary Figure 4. Replication kinetics or recombinant VSV pseudotyped with EBOV or MARV glycoprotein
Supplementary Figure 5. EBOV and MARV antagonize the IFN-I response with similar efficiencies in ERB cells
Supplementary Figure 6. EBOV and MARV efficiently antagonize human IFN-I signaling
Supplementary Figure 7. Validation of Chiroptera IFNβ and IFN-I inhibitors in JFB cells
Supplementary Figure 8. Representative gating strategy for flow cytometric analysis of splenocytes

Supplementary Table 1. Hematology of EBOV and MARV infected JFBs

Study Day	Animal ID	RBC (M/uL)	WBC (K/uL)	HGB (g/dL)	NEUT# (K/uL)	NEUT% (%)	HCT (%)	LYMPH# (K/uL)	LYMPH% (%)	MCV (fL)	MONO# (K/uL)	MONO% (%)	MCH (pg)	EOS# (K/uL)	EOS% (%)	MCHC (g/dL)	BASO# (K/uL)	BASO% (%)	RDW SD (fL)	RDW CV (%)	PLT (K/uL)	MPV (fL)	RET# (K/uL)	RET% (%)
-7	EBO01	14.36	10.94	19.6	5.02	45.9	48.9	5.15	47.1	34.1	0	0	13.6	0	0	40.1	0.77	7	31	37.1	456	9.3	110.6	0.77
3	EBO01	13.63	5.19	18.9	2.68	51.6	46.6	2.22	42.8	34.2	0.05	1	13.9	0	0	40.6	0.24	4.6	29.3	35.6	752	12	205.8	1.51
-8	EBO02	12.96	5.73	17.7	3.06	53.4	42.5	2.67	46.6	32.8	0	0	13.7	0	0	41.6	0	0	27.1	33.8	420	10.6	134.8	1.04
3	EBO02	12.23	5.29	16.7	1.86	35.2	41.8	2.88	54.4	34.2	0.04	0.8	13.7	0	0	40	0.51	9.6	26.2	31.3	448	13	332.7	2.72
-8	EBO07	12.93	10.45	17.5	1.78	17	43	8.42	80.6	33.3	0.01	0.1	13.5	0	0	40.7	0.24	2.3	26.6	33.5	425	9.7	250.8	1.94
3	EBO07	13.25	7.16	17.5	0.49	6.8	44.5	6.52	91.1	33.6	0.06	0.8	13.2	0	0	39.3	0.09	1.3	26.1	32.8	613	12	382.9	2.89
-8	EBO08	13.89	10.99	18.7	8.5	77.3	43.4	2.22	20.2	31.2	0.01	0.1	13.5	0	0	43.1	0.26	2.4	26.2	37.9	521	8.8	123.6	0.89
3	EBO08	12.34	7.6	16.4	5.35	70.4	39.5	2.07	27.2	32	0.09	1.2	13.3	0.01	0.1	41.5	0.08	1.1	24.9	33.4	451	12.9	246.8	2
-8	EBO03	14.52	3.03	20.2	2.34	77.2	49.5	0.69	22.8	34.1	0	0	13.9	0	0	40.8	0	0	29.4	36.8	547	10.8	88.6	0.61
7	EBO03	12.07	10.21	16.5	7.02	68.7	41.2	3.14	30.8	34.1	0.05	0.5	13.7	0	0	40	0	0	25.6	30.7	799	12.1	144.8	1.2
-7	EBO04	13.42	10.56	19.2	2.56	24.2	49.1	8	75.8	36.6	0	0	14.3	0	0	39.1	0	0	26.7	30.7	537	10.4	95.3	0.71
7	EBO04	12.78	14.18	18.3	8.42	59.4	47.5	5.56	39.2	37.2	0.09	0.6	14.3	0	0	38.5	0.11	0.8	26.5	30	733	11.1	337.4	2.64
-8	EBO09	13.93	5.18	18.6	2.36	45.6	46.6	2.82	54.4	33.5	0	0	13.4	0	0	39.9	0	0	26.8	34.3	889	9.5	79.4	0.57
7	EBO09	12.45	10.83	16.4	4.77	44	41	5.8	53.6	32.9	0.02	0.2	13.2	0	0	40	0.24	2.2	24.8	31.3	902	11.1	146.9	1.18
-8	EBO10	14.12	4.85	18.7	2.69	55.5	47.1	2.16	44.5	33.4	0	0	13.2	0	0	39.7	0	0	28.3	35.8	540	9.1	89	0.63
7	EBO10	12.91	12.72	16.9	3.89	30.6	42.6	8.75	68.8	33	0.08	0.6	13.1	0	0	39.7	0	0	27.1	33.3	772	11.8	227.2	1.76
-8	EBO05	13.76	4.63	18.9	2.14	46.2	45.1	2.37	51.2	32.8	0	0	13.7	0	0	41.9	0.12	2.6	27.8	36.4	33	6.7	60.5	0.44
28	EBO05	12.91	6.97	17.2	2.87	41.2	42.3	4.09	58.7	32.8	0.01	0.1	13.3	0	0	40.7	0	0	26.5	33.5	943	9.9	370.5	2.87
-8	EBO06	14.8	5.78	20.1	2.86	49.5	47.6	2.6	45	32.2	0	0	13.6	0	0	42.2	0.32	5.5	28.9	39.4	511	8.9	97.7	0.66
28	EBO06	13.47	5.63	17.8	1.5	26.6	44.4	4.04	71.8	33	0.02	0.4	13.2	0	0	40.1	0.07	1.2	27.7	34.9	950	10.6	281.5	2.09
-8	EBO11	11.74	11.51	15.9	9.42	81.8	40.8	2.08	18.1	34.8	0.01	0.1	13.5	0	0	39	0	0	25	29.3	816	10.5	179.6	1.53
28	EBO11	11.84	12.45	16.4	4.63	37.1	42.1	7.8	62.7	35.6	0.02	0.2	13.9	0	0	39	0	0	26	29	1163	11.3	440.4	3.72
-8	EBO12	12.57	8.31	18.4	1.66	20	47.4	6.65	80	37.7	0	0	14.6	0	0	38.8	0	0	25.7	28.4	790	10.1	206.1	1.64
28	EBO12	11.97	10.03	17.3	2.01	20	43.9	7.75	77.3	36.7	0.01	0.1	14.5	0	0	39.4	0.26	2.6	25.1	28.1	621	12.2	397.4	3.32
-7	MARV01	14.05	3.32	19.3	1.93	58.1	47.3	1.39	41.9	33.7	0	0	13.7	0	0	40.8	0	0	27.1	34.9	521	10.2	61.8	0.44
3	MARV01	11.98	3.02	16.4	1.33	44	41.6	1.67	55.3	34.7	0.02	0.7	13.7	0	0	39.4	0	0	24.3	28.6	684	12	135.4	1.13
-7	MARV02	2.49	----	3.3	----	----	6.2	----	----	24.9	----	----	13.3	----	----	53.2	----	----	20.9	22.4	81	9.8	15.2	0.61
3	MARV02	13.47	5.36	18	1.96	36.5	44.4	3.22	60.1	33	0.02	0.4	13.4	0	0	40.5	0.16	3	27.7	34.7	725	11.4	255.9	1.9
-7	MARV07	12.54	24	17.4	5.64	23.4	39.7	16.72	69.7	31.7	0.02	0.1	13.9	0	0	43.8	1.62	6.8	24.1	34.7	333	8.8	267.1	2.13
3	MARV07	13.61	20.56	18.3	4.27	20.9	44.1	16.06	78.1	32.4	0.17	0.8	13.4	0.01	0	41.5	0.05	0.2	24.8	34.3	221	10.3	371.6	2.73
-7	MARV08	13.26	10.23	17.5	5.41	52.9	42.6	4.82	47.1	32.1	0	0	13.2	0	0	41.1	0	0	26.3	35.4	496	9.6	110.1	0.83
3	MARV08	11.82	3.37	15.7	1.88	55.8	39.4	1.43	42.4	33.3	0.01	0.3	13.3	0	0	39.8	0.05	1.5	27.2	31.6	604	10.7	450.3	3.81
-7	MARV03	12.98	7.45	18	3.93	52.8	44.1	3.52	47.2	34	0	0	13.9	0	0	40.8	0	0	26.4	32.5	631	10.3	106.4	0.82
7	MARV03	11.77	6.98	16.1	3.05	43.6	40.6	3.85	55.2	34.5	0.04	0.6	13.7	0	0	39.7	0.04	0.6	24.5	29.2	834	12.2	258.9	2.2
-7	MARV04	12.94	6.49	18.7	3.28	50.6	44.5	2.85	43.9	34.4	0	0	14.5	0	0	42	0.36	5.5	26	32.1	635	9.5	116.5	0.9
7	MARV04	12.24	3.87	17.3	1.44	37.2	44.1	2.43	62.8	36	0	0	14.1	0	0	39.2	0	0	26.1	28.9	991	10.7	308.4	2.52
-7	MARV09	13.83	14.17	19.4	4.67	32.9	47.7	8.68	61.3	34.5	0.01	0.1	14	0	0	40.7	0.81	5.7	28.9	34.6	812	10	113.4	0.82
7	MARV09	12.93	15.42	18.2	9.85	63.9	45	5.51	35.7	34.8	0.06	0.4	14.1	0	0	40.4	0	0	27.9	32.1	1065	11.5	350.4	2.71
-7	MARV10	14.86	5.91	19.3	2.52	42.7	45.6	3.14	53.1	30.7	0	0	13	0	0	42.3	0.25	4.2	28.2	40.4	574	9.5	173.9	1.17
7	MARV10	14.49	9.65	18.7	7.92	82.1	45.2	1.71	17.7	31.2	0.02	0.2	12.9	0	0	41.4	0	0	28.2	38.9	857	10.2	336.2	2.32
-7	MARV05	12.03	3.01	17.3	1.63	54.2	43.7	1.18	39.2	36.3	0	0	14.4	0	0	39.6	0.2	6.6	26.3	28.5	446	8.9	156.4	1.3

28	MARV05	11.8	3.78	17.6	0.96	25.4	46.2	2.8	74.1	39.2	0.02	0.5	14.9	0	0	38.1	0	0	27.8	27.4	781	10.3	472	4
-7	MARV06	12.26	3.22	17.1	1.74	54	41.5	1.48	46	33.8	0	0	13.9	0	0	41.2	0	0	26.5	31.2	530	10.9	83.4	0.68
28	MARV06	11.94	4.93	16.3	2.68	54.4	42.5	2.18	44.2	35.6	0.03	0.6	13.7	0	0	38.4	0.04	0.8	26	29.4	464	13.5	401.2	3.36
-7	MARV11	13.43	8.97	18.8	4.66	52	48.7	4.31	48	36.3	0	0	14	0	0	38.6	0	0	27.2	31	728	11.3	192	1.43
28	MARV11	13.23	16.15	18	1.58	9.8	47.4	14.53	90	35.8	0.04	0.2	13.6	0	0	38	0	0	27.6	31.5	744	12	654.9	4.95
-7	MARV12	13.94	18.77	18.3	6.25	33.3	45.4	12.36	65.8	32.6	0.16	0.9	13.1	0	0	40.3	0	0	26.9	36	502	11.2	111.5	0.8
28	MARV12	13	6.04	17.1	1.04	17.2	43.7	4.93	81.6	33.6	0.02	0.3	13.2	0.01	0.2	39.1	0.04	0.7	29.2	34.5	820	11.2	434.2	3.34

red blood cells (RBC), white blood cells (WBC), hemoglobin (HGB), neutrophil (NEUT), hematocrit (HCT), lymphocytes (LYMPH), mean corpuscular volume (MCV), monocytes (MONO), mean corpuscular hemoglobin (MCH), eosinophils (EOS), mean corpuscular hemoglobin concentration (MCHC), basophils (BASO), red cell distribution width (RDW-SD), red cell distribution width-coefficient of variation (RDW CV), platelets (PLT), mean platelet volume (MPV), reticulocytes (RET), number (#), percentage (%)

Supplementary Table 2. Clinical chemistry of healthy control and EBOV or MARV-infected JFBs

Study Day	Animal ID	BUN (mg/dL)	Creatinine (mg/dL)	ALT (U/L)	ALP (U/L)	AST (U/L)	T Bilirubin (mg/dL)	Glucose (mg/dL)	Calcium (mg/dL)	T Protein (g/dL)	Albumin (g/dL)	Globulin (g/dL)	Na+ (mmol/L)	K+ (mmol/L)	CL- (mmol/L)	tC02 (mmol/L)	Hem	Lip	Notes
Healthy Control	BT01	9	0.4	83	106	188	0.3	53	9.5	6.4	3.0	3.4	145	3.8	108	23	0	0	
Healthy Control	BT02	9	0.2	64	139	106	0.2	130	10.5	6.0	3.1	2.9	146	4.6	106	22	0	0	
Healthy Control	BT03	9	0.4	66	101	235	0.2	175	9.9	6.4	3.4	3.0	138	3.9	103	24	0	0	
Healthy Control	BT04	10	0.3	69	98	128	0.3	157	9.5	6.5	3.4	3.1	144	4.8	106	26	0	0	
Healthy Control	BT05	4	0.4	76	146	125	0.2	76	10.2	6.2	3.2	3.0	145	3.9	100	24	0	0	
Healthy Control	BT06	6	0.2	80	76	193	0.2	61	9.4	5.8	2.9	2.9	143	4.6	108	23	0	0	
Healthy Control	BT07	10	0.6	135	300	283	0.2	89	9.9	5.5	2.7	2.8	145	5.9	113	24	0	0	
Healthy Control	BT08	7	0.3	65	72	172	0.2	158	9.9	6.1	2.6	3.5	138	4.3	106	22	0	0	
Healthy Control	BT09	5	0.2	51	48	179	0.2	134	9.1	5.6	2.4	3.2	142	5.1	110	18	0	0	
Healthy Control	BT10	5	<0.2	96	128	244	0.2	151	10.0	5.6	2.9	2.7	139	5.5	105	20	0	0	
3	EBO01	7	0.4	85	60	306	0.2	126	8.8	5.8	3.1	2.8	150.0	6.3	105	23	2	0	
3	EBO02	9	0.2	103	106	238	0.2	138	9.6	6.0	3.1	2.8	151	8.5	107	16	0	0	
3	EBO07	11	<0.2	55	83	173	0.3	111	8.1	5.8	3.1	2.7	146	6.2	103	26	0	0	
7	EBO08	8	0.2	54	72	115	0.2	160	9.3	6.2	3.1	3.1	151.0	7.4	107	22	0	0	
7	EBO03																		Insufficient sample
7	EBO04																		Insufficient sample
7	EBO09	8	0.3	65	71	122	0.2	290	8.6	6.0	3.0	3.1	142	>8.5	99	14	0	0	
28	EBO10	8	<0.2	59	84	111	0.1	200	10.5	6.9	3.4	3.5	150.0	8.4	107	22	0	0	
28	EBO05																		Insufficient sample
28	EBO06	8	<0.2	51	78	98	0.2	191	9.3	6.1	3.0	3.1	144	6.2	107	26	0	0	
28	EBO11	7	0.2	37	65	81	<0.1	226	10.7	6.3	3.3	3.0	145	>8.5	108	25	0	0	
3	EBO12																		Insufficient sample
3	MARV01																		Insufficient sample
3	MARV02	7	<0.2	71	106	397	0.2	140	9.3	6.0	3.0	3.0	146	6.2	106	18	1	0	
3	MARV07	12	<0.2	87	65	191	0.2	150	9.4	5.8	3.1	2.7	143	6.0	101	23	1	0	
7	MARV08	8	0.2	61	67	149	0.2	202	8.6	6.1	3.2	2.9	142	5.8	103	20	0	0	
7	MARV03	7	0.4	46	56	135	0.3	189	9.4	6.7	3.6	3.0	145	7.6	100	21	0	0	
7	MARV04	7	0.3	44	97	102	0.2	305	10.3	5.9	3.0	2.9	146	>8.5	97	23	0	0	
7	MARV09	10	<0.2	43	67	90	0.2	192	9.7	6.4	3.6	2.8	149	>8.5	104	19	2	0	
28	MARV10																		Insufficient sample
28	MARV05	6	0.3	55	97	132	0.1	243	10.4	6.5	3.3	3.2	147	>8.5	105	18	2	0	
28	MARV06	8	0.2	48	115	201	0.1	347	10.6	5.9	2.9	3.0	145	>8.5	103	24	0	0	
28	MARV11	10	<0.2	71	67	113	0.1	163	9.1	6.1	3.2	2.9	146	6.7	107	24	0	0	

Blood urea nitrogen (BUN), alanine aminotransferase (ALT), alkaline phosphatase (ALP), aspartate aminotransferase (AST), sodium (Na+), potassium (K+), chlorine (Cl-), total carbon dioxide (tCO2), heme (Hem), lipase (Lip),

Supplementary Table 3. Differentially Upregulated genes in EBOV or MARV-infected JFBs compared to healthy controls. Gene ontology biological processes enrichment results for each comparison. Input genes were those FDR<0.1 and with at least 2-fold changes in either direction. Statistical significance was tested with a Fisher's exact test.

Challenge Virus	Direction	adj.Pval	nGenes	Pathways
EBOV	Up regulated	9.30E-63	176	Immune response
	Up regulated	1.19E-56	199	Immune system process
	Up regulated	7.37E-50	136	Defense response
	Up regulated	2.30E-42	121	Response to biotic stimulus
	Up regulated	8.84E-42	118	Response to other organism
	Up regulated	2.03E-40	124	Biological process involved in interspecies interaction between organisms
	Up regulated	8.79E-40	100	Defense response to other organism
	Up regulated	1.26E-39	91	Innate immune response
	Up regulated	1.28E-38	125	Regulation of immune system process
MARV	Up regulated	4.47E-27	58	Immune response
	Up regulated	4.23E-26	50	Defense response
	Up regulated	2.85E-25	64	Immune system process
	Up regulated	3.42E-24	48	Biological process involved in interspecies interaction between organisms
	Up regulated	4.25E-23	37	Innate immune response
	Up regulated	5.13E-23	45	Response to biotic stimulus
	Up regulated	9.83E-23	44	Response to other organism
	Up regulated	1.78E-22	39	Defense response to other organism
	Up regulated	1.62E-18	43	Regulation of immune system process

Supplementary Table 4. Differentially Upregulated genes in EBOV-infected compared to MARV-infected JFBs. Gene ontology biological processes enrichment results for each comparison. Input genes were those FDR<0.1 and with at least 2-fold changes in either direction. Statistical significance was tested with a Fisher's exact test.

Direction	adj.Pval	nGenes	Pathways	Genes
Up regulated	0.00011027	4	Negative regulation of viral process	RSAD2 PML ISG15 LY6E
Up regulated	0.00011027	4	Type I interferon signaling pathway	RSAD2 ISG15 PTPN1 PSMB8
Up regulated	0.00011027	4	Cellular response to type I interferon	RSAD2 ISG15 PTPN1 PSMB8
Up regulated	0.00046032	2	Cytosol to endoplasmic reticulum transport	TAP2 TAP1
Up regulated	0.00046032	4	Regulation of viral life cycle	RSAD2 PML ISG15 LY6E
Up regulated	0.000846493	5	Regulation of response to biotic stimulus	IL4I1 PML ISG15 PTPN1 PSMB8
Up regulated	0.000846493	9	Immune response	IL4I1 RSAD2 IFI44L PML ISG15 PTPN1 TAP2 PSMB8 TAP1
Up regulated	0.000846493	4	Regulation of viral process	RSAD2 PML ISG15 LY6E
Up regulated	0.000846493	2	Vesicle fusion with endoplasmic reticulum-Golgi intermediate compartment (ERGIC) membrane	TAP2 TAP1
Up regulated	0.000939666	7	Viral process	RSAD2 PML ISG15 TAP2 PSMB8 TAP1 LY6E
Up regulated	0.001179496	3	Antigen processing and presentation of exogenous peptide antigen via MHC class I, TAP-dependent	TAP2 PSMB8 TAP1
Up regulated	0.001179496	2	Antigen processing and presentation of endogenous peptide antigen	TAP2 TAP1
Up regulated	0.001201765	4	Defense response to virus	RSAD2 IFI44L PML ISG15
Up regulated	0.001458967	7	Response to biotic stimulus	IL4I1 RSAD2 IFI44L PML ISG15 PTPN1 PSMB8
Up regulated	0.001905862	6	Defense response to other organism	RSAD2 IFI44L PML ISG15 PTPN1 PSMB8

Supplementary Table 5. RT-qPCR primers for JFB and ERB immune genes and viruses

Target	Sequence
EBOV F	CAGCCAGCAATTTCTTCCAT
EBOV R	TTTTCGGTTGCTGTTTCTGTG
EBOV P	FAM-TCATTGGCGTACTGGAGGAGCAGG
MARV F	GCAAAAGCATTCCCTAGTAACATGA
MARV R	CACCCCTCACTATRGCGTTYTC
MARV P	FAM-TGGCACCAYAATTCAGCAAGCATAGG
JFB *Ifnb1* F	ACTTCAAGTTTCCCGAGGAGA
JFB *Ifnb1* P	FAM-GCACGGGCTGGAATGAGACCATCATTGA
JFB *Ifnb1* R	GGTCCATCTGCCAACTGAGT
JFB *Isg15* F	CAGAAGGTGGCTGAGCTGAA
JFB *Isg15* P	FAM-TGGCTGAGTTTCCAGGGGAGGCCC
JFB *Isg15* R	CTTGTATTCCTTCAGCTGCGC
JFB *Ifit1* F	AGAGCTTGAAGCAGGCTGAA
JFB *Ifit1* P	FAM-ACATGCTGGCCAGTCGGAGGTGAG
JFB *Ifit1* R	CTTCTAGTCTGCCCATGCGG
JFB *Mx1* F	GTTCTTCATGCTCCGGTCGT
JFB *Mx1* P	FAM-GCCAGAAGCTGAGCAATGCCATGTTGC
JFB *Mx1* R	TTCCTCTTGTCGCTGGTGTC
JFB *Oas1* F	CGTGGTGCAGAGTCACAGAT
JFB *Oas1* P	FAM-CGCGTGATACGGAAACCTCGCTCGC
JFB *Oas1* R	GGGCAGGACATCAAACTCCA
JFB *Il6* F	AACAGCAAGGAGGCACTGAC
JFB *Il6* P	FAM-ACCTGAACCTTCCGAAACTGACAAGAAG
JFB *Il6* R	CAGACCGGTGGTGAGTCTC
JFB *Tnfa* F	ACTGGCTCAGACCCTTGGAT
JFB *Tnfa* P	FAM-ACCCCAAGTGACAAGCCTGTTGCCC
JFB *Tnfa* R	GAGAGCATTGGCCACCTGAT
ERB *Ifnb1* F	TCCACCACAGCTCTTTCCATGA
ERB *Ifnb1* P	FAM-CCGCCACAGGAGCTTCAGGCAGGC
ERB *Ifnb1* R	AGTCCATCCTGTCCTTGAGGCAA
ERB *Isg15* F	GAGTTCCTGGTGCCCGTGAC
ERB *Isg15* P	FAM-TGCCCTGCTTCCGGCAGCGCC
ERB *Isg15* R	CCTGCAGCACCTTGCTGTCC
ERB *Ifit1* F	ACAGGCTGGAGCAGCTGAGA
ERB *Ifit1* P	FAM-CGTGGGAGTGCACAACCTACTGGCC
ERB *Ifit1* R	CAAGCTCTCCAGGGCTTCCT
ERB *Mx1* F	AGCATGGCTCAGGAGGTGGA
ERB *Mx1* P	FAM-TGCCCTTGTCCACCAGGTCGGGCT
ERB *Mx1* R	GTCCACGACCCTGTGTTCGG
ERB *Oas1* F	TGGCGGTGGAGACTCACAGA
ERB *Oas1* P	FAM-AGCCTCTCGCGCCAGCCGCG
ERB *Oas1* R	ACCAGGAGCCCACTGGAGAG
ERB *Il6* F	GGTCCAGGTGCTGAAGCAGA
ERB *Il6* P	FAM-TGCAGCCACTCGCTCTGCGACTGC
ERB *Il6* R	GGCTTCGCAGGATGAGGTGA
ERB *Tnfa* F	CTGTCGCCCACGTTGTAGCA
ERB *Tnfa* P	FAM-ACAGCTCCAGTGGCTGAGCCAGCGT
ERB *Tnfa* R	TTGGCCAGGAGAGCATTGGC
AJ *Hprt* F	AGATGGTGAAGGTCGCAAG
ERB *Hprt* F	AGATGGTCAAGGTCGCAAG
AJ/ERB *Hprt* P	FAM-ACTTTGTTGGATTTGAAATTCCAGACAAGTTTG
AJ/ERB *Hprt* R	CCTGAAGTATTCATTATAGTCAAGGG
AJ IgM	GAAGTCCTTGGCCAGGCAGCCCACAGT
AJ IgG	CAGGACACAGTCACCGGCTCAGGGAAG

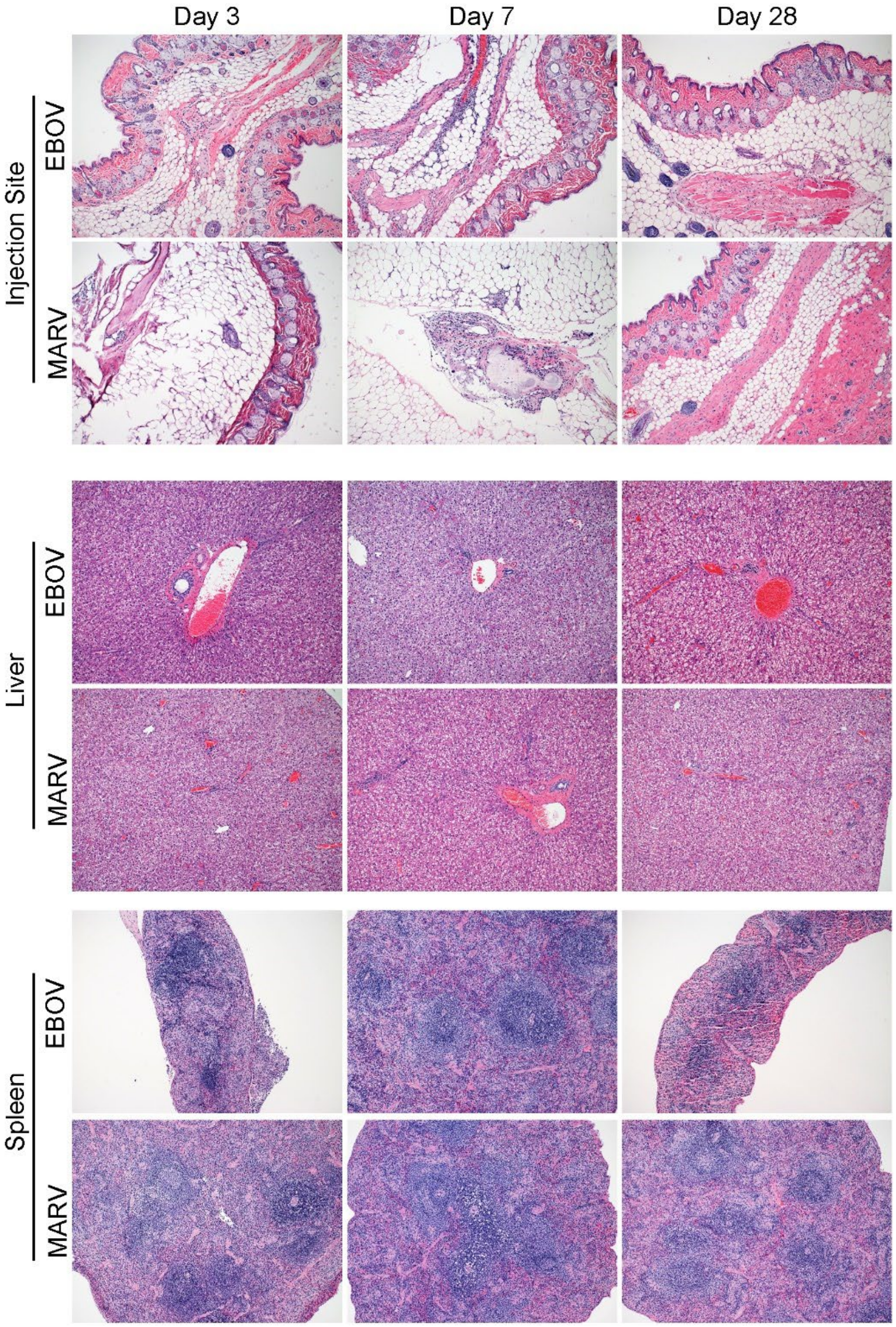

Supplementary Figure 1. Infection with EBOV or MARV does not induce histopathologic changes in JFBs

Tissue sections were stained with hematoxylin and eosin. Representative sections for skin at the injection site, liver, and spleen sections for Ebola virus (EBOV) or Marburg virus (MARV) challenged bats at 3, 7, and 28 days post-inoculation. The following magnification was used for all slides: 100X.

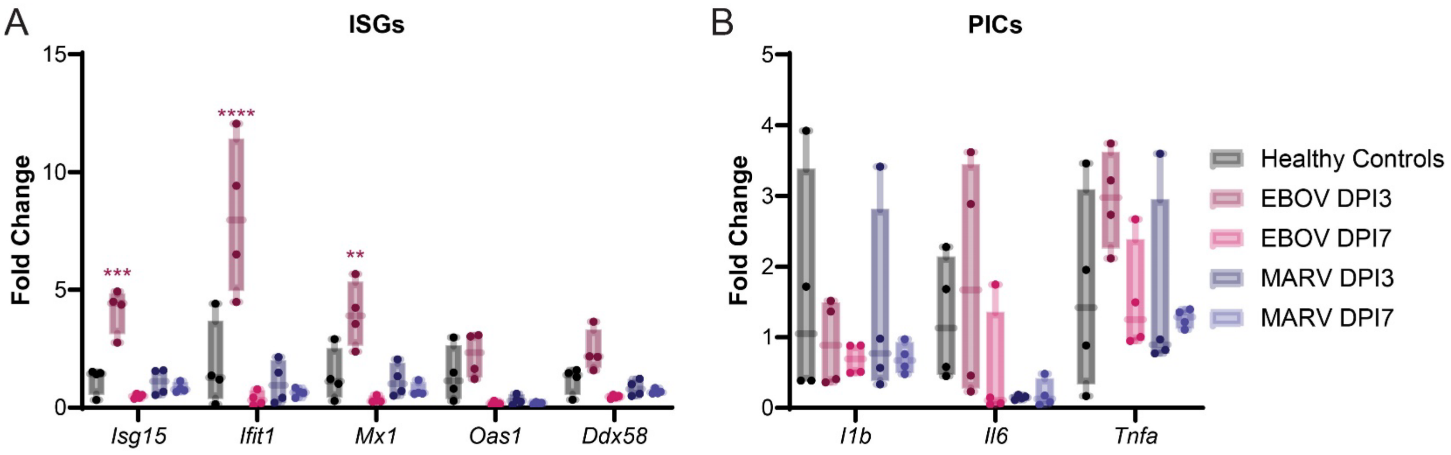

Supplementary Figure 2. EBOV infection induces mild ISG expression in the lung

RT-qPCR of interferon-stimulate genes (ISGs) (A) or pro-inflammatory cytokines (PICs) (B) mRNA in lungs collected from healthy control bats (n=4) and at day (D) 3 or 7 necropsy of EBOV- or MARV-infected bats (n=4). (A-B) ΔC_T values were normalized to the average ΔC_T value of the healthy control bats to calculate $\Delta\Delta C_T$. Fold change of each gene at necropsy day was compared to the healthy controls using a two-way ANOVA with Dunnett's multiple comparison. Box plots indicate median (middle line), 25th, 75th percentile (box) as well as outliers (single points).

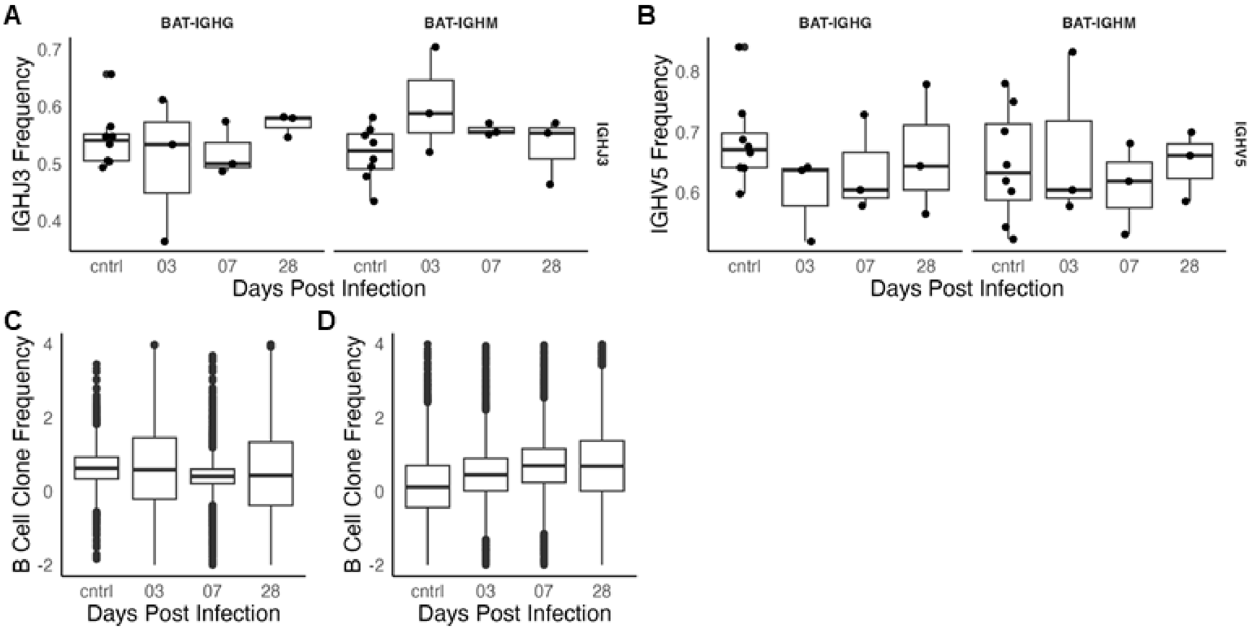

Supplementary Figure 3. BCR sequencing

(A) Clonal frequencies of IgG sequences in the spleens of healthy control bats (n=10) or Ebola virus challenged bats at day 3, 7, and 28 necropsies (n=3). (B) Clonal frequencies of IgM BCR sequences. (A-B) Data represents a sample from the posterior effect estimates obtained from a Bayesian hierarchical model. Points represent the frequency observed in individual bats. Boxplots show the 25th, 50th (median), and 75th percentiles, with lines indicating the smallest and largest values within 1.5 times the interquartile range. (C) Frequency of BCR sequences containing the putative IGHV5 (C) or IGHJ3 (D) gene segment. Dots represent data from individual bats.

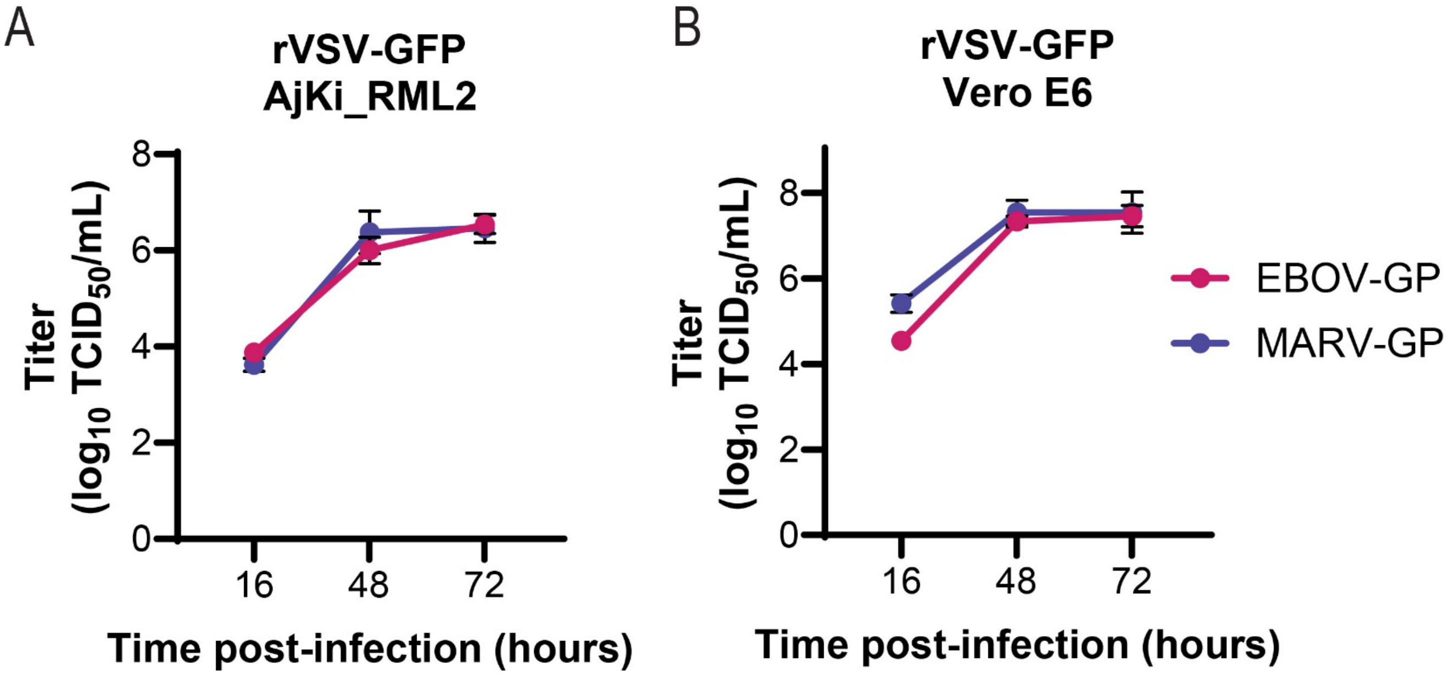

Supplementary Figure 4. Replication kinetics of recombinant VSV expressing EBOV or MARV GP

Infectious titers of rVSV-EBOV-GFP or rVSV-MARV-GFP from AjKi-RML2 (A) or Vero E6 (B) cells infected at multiplicity of infection (MOI) 0.005 at 16, 48, and 72 hours post-infection. (A-B) Two independent experiments were performed with 3 technical replicates for each experiment. The titer at each time point was compared using a two-way ANOVA with Dunnett's multiple comparison. The data is presented as the mean +/- SD.

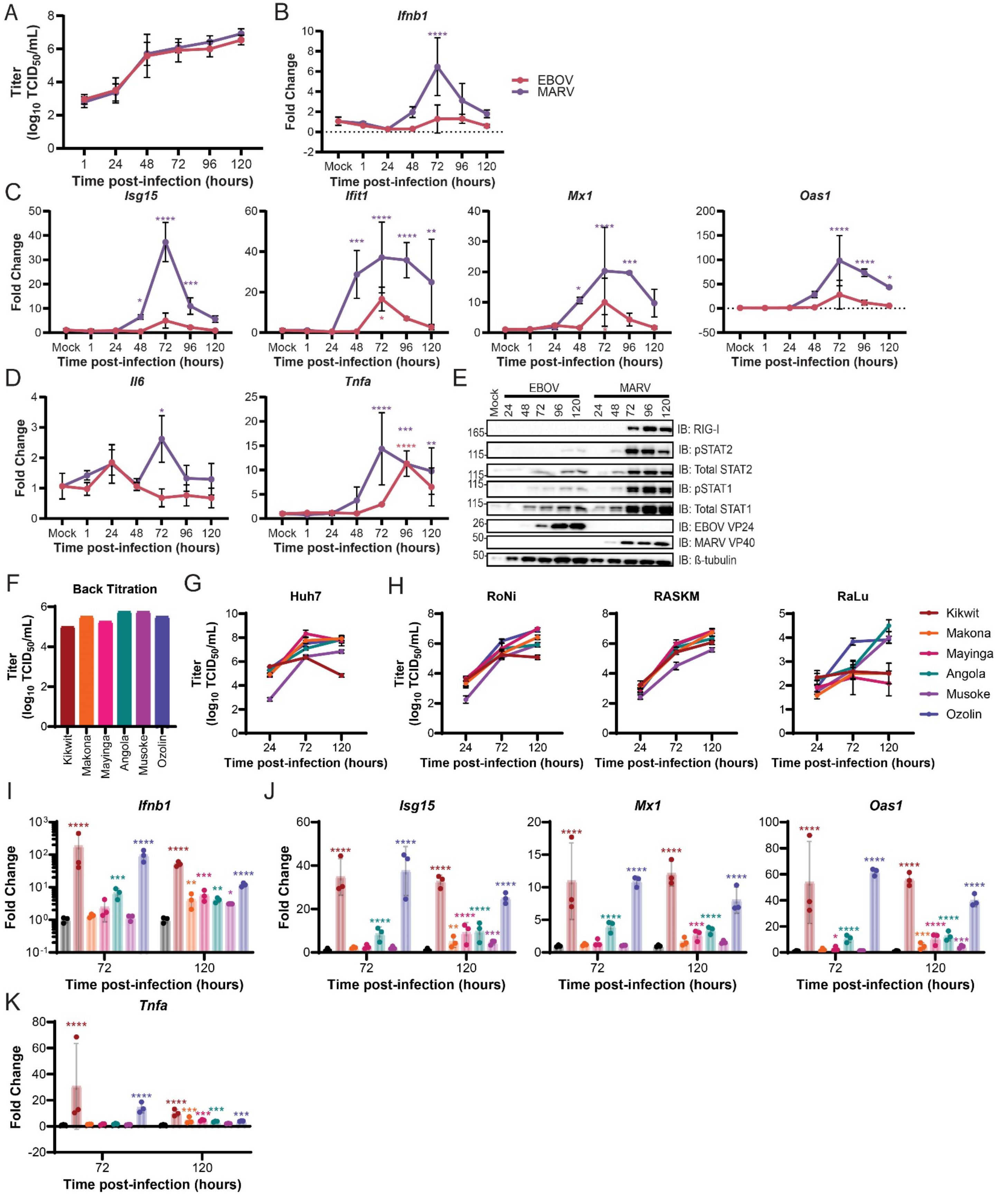

A
Titer (log10 TCID50/mL)
Time post-infection (hours)
B
Ifnb1
Fold Change
Time post-infection (hours)
EBOV
MARV
C
Isg15
Ifit1
Mx1
Oas1
D
Il6
Tnfa
E
EBOV
MARV
Mock
IB: RIG-I
IB: pSTAT2
IB: Total STAT2
IB: pSTAT1
IB: Total STAT1
IB: EBOV VP24
IB: MARV VP40
IB: ß-tubulin
F
Back Titration
Kikwit
Makona
Mayinga
Angola
Musoke
Ozolin
G
Huh7
H
RoNi
RASKM
RaLu
I
Ifnb1
J
Isg15
Mx1
Oas1
K
Tnfa

Supplementary Figure 5. EBOV and MARV antagonize the IFN-I response with similar efficiencies in ERB cells

(A) Infectious titers of EBOV-Mayinga or MARV-Ozolin on Egyptian rousette bat kidney cells (RoNi) infected with multiplicity of infection (MOI) 0.1. Data presented as $\log_{10}$ transformed values. Data is from two independent experiments with three technical replicates per experiment. Two-way ANOVA with Sidak's multiple test comparison was performed to evaluate the difference between EBOV and MARV titer at each time point. RT-qPCR of *Ifnb1* (B), interferon stimulated gene (C), or pro-inflammatory cytokine (D) mRNA in RoNi cells infected with EBOV or MARV at MOI 0.1. (E) Immunoblot of RoNi cells infected with EBOV or MARV at MOI 0.1. The presented panel is representative of three independent immunoblots. (F) Infectious titers of inoculum of the three EBOV and MARV strains used to infect Huh7 (S5G), AjKi_RML2 (6F), and Egyptian rousette cells (S5H) to assess replication kinetics. This data is from one replicate. (G) Infectious titers of three EBOV strains and three MARV strains on human hepatoma (Huh7), cells infected with MOI 0.1. Data presented as $\log_{10}$ transformed values. Data is from one experiment conducted in triplicate. (H) Infectious titers of three EBOV strains and three MARV strains on Egyptian rousette bat immortalize kidney (RoNi), primary kidney (RASKM), and primary lung (RaLu) cells infected with MOI 0.1. Data presented as $\log_{10}$ transformed values. Data is from one experiment conducted in triplicate. RT-qPCR of *Ifnb1* (I), interferon stimulated gene (J), or *Tnfa* (K) mRNA in RoNi cells infected with three different EBOV or MARV strains at MOI 0.1. (B-D;I-K) ΔC_T values were normalized to the average ΔC_T value of mock infected cells to calculate $\Delta\Delta C_T$. Data from three biological replicates. Fold change of each gene at each time point for EBOV and MARV-infected cells was compared mock using a two-way ANOVA with Dunnett's multiple test comparison. The data is presented as the mean +/- SD. P-values for comparisons to mock cells are indicated. **** <0.0001, *** <0.001, ** < 0.01, * <0.05.

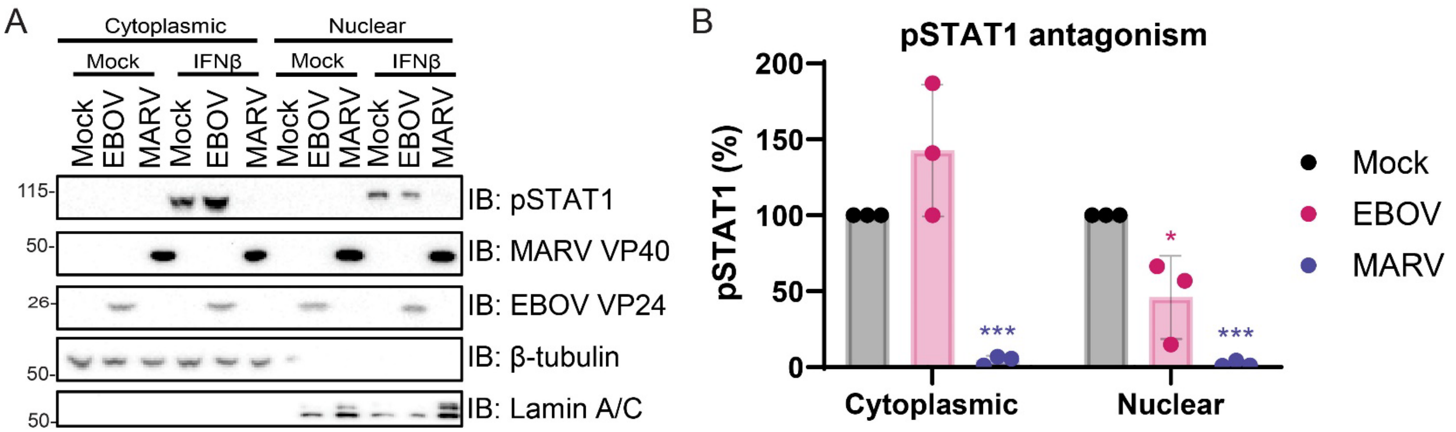

Supplementary Figure 6. EBOV and MARV efficiently antagonize human IFN-I signaling

(A) Immunoblot of cytoplasmic and nuclear fractions of human hepatoma (Huh7) cells infected with MOI 1.5 of EBOV-Mayinga or MARV-Ozolin for 24 hours prior to the addition of exogenous recombinant Chiroptera IFN-β (100ng/mL) for 30 minutes. The presented panel is representative of three independent immunoblots. (B) Quantification of phosphorylated STAT1 in the cytoplasmic and nuclear fractions. Normalized expression was determined using the following formula: (AUC pSTAT1)/(AUC β-tubulin (cytoplasmic) or Lamin A/C (nuclear)). To calculate relative expression, the normalized sample value is divided by the normalized expression in uninfected cells treated with IFN-β. Three independent experiments were performed. Percentage of pSTAT1 in each of the fractions for EBOV- and MARV-infected cells was compared mock using a two-way ANOVA with Dunnett's multiple test comparison. The data is presented as the mean +/- SD. P-values for comparisons to mock cells are indicated. *** <0.001, * <0.05.

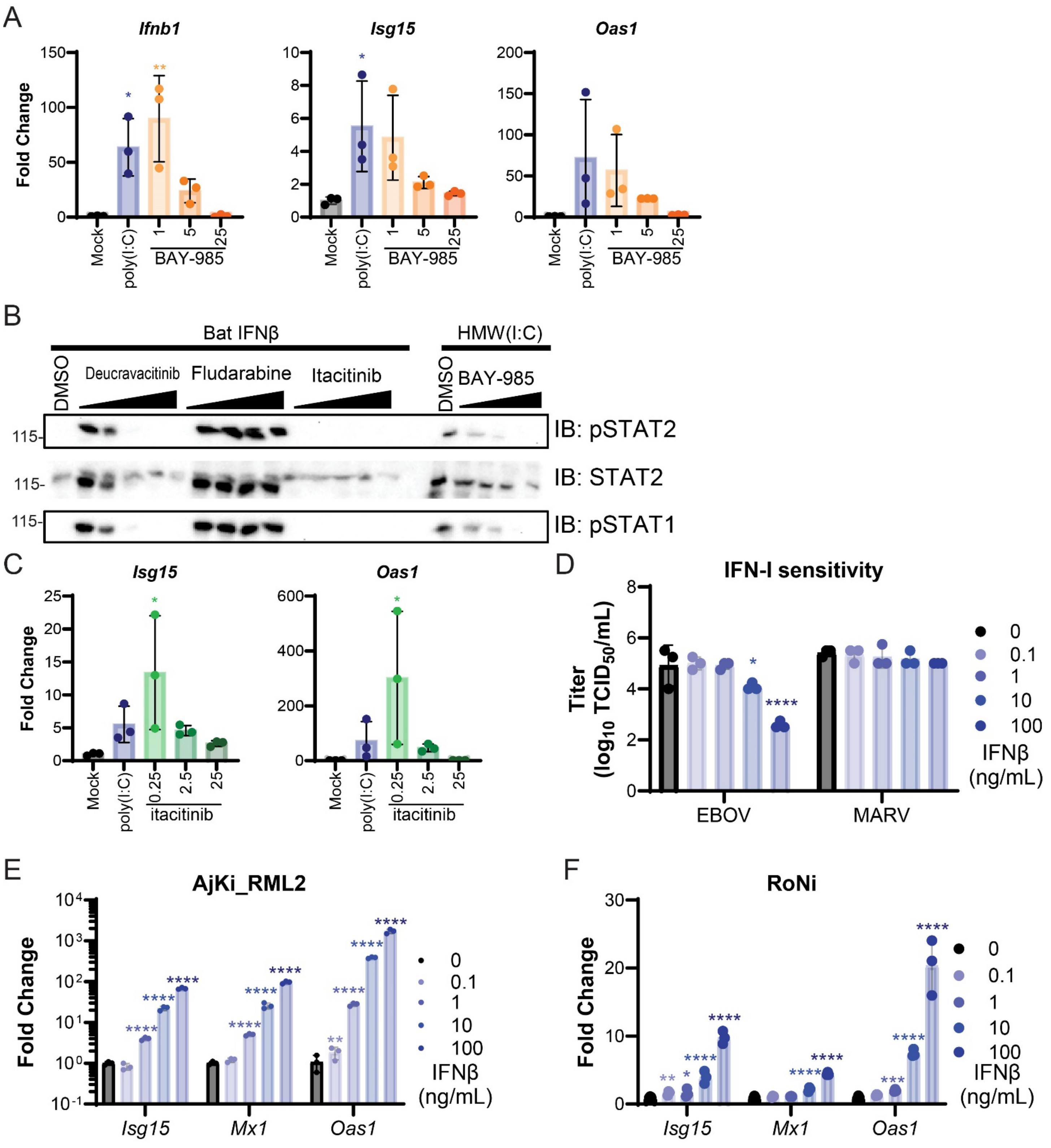

Supplementary Figure 7. Validation of Chiroptera IFN-β and IFN-I inhibitors in JFB cells

(A) RT-qPCR of *Ifnb1* or interferon-stimulate gene (ISG), *Isg15* and *Oas1*, mRNAs in Jamaican fruit bat kidney cells, AjKi_RML2, were treated with type I interferon induction inhibitor BAY-985 prior to transfection of high molecular weight poly(I:C). (B) Immunoblot of AjKi_RML2 cells treated with IFN-I signaling (deucravacitinib, fludarabine, or itacitinib) or induction (BAY-985) inhibitors prior to addition of Chiroptera IFN-β for 30 minutes (IFN-I signaling inhibitors) or transfection of high molecular weight poly(I:C) for 24 hours (BAY-985). (C) RT-qPCR of *Isg15* and *Oas1*, mRNAs in AjKi_RML2 cells treated with type I signaling inhibitor itacitinib prior to transfection of high molecular weight poly(I:C). (A-C) ΔC_T (n=3) values were normalized to the average ΔC_T value of mock infected cells to calculate $\Delta\Delta C_T$. Fold change of each gene at dose was compared to unstimulated cells treated with DMSO using a one-way ANOVA with Dunnett's multiple test comparison. (D) Infectious titers of EBOV-Mayinga or MARV-Ozolin at 48 hours post-infection. RoNi cells were treated with recombinant Chiroptera IFN-β for 24 hours prior to infection with MOI 0.1. Data presented as $\log_{10}$ transformed values. The experiment was performed in triplicate. To test a change in viral titer at each dose compared to mock treated cells, we performed a two-way ANOVA with Dunnett's multiple test comparison. RT-qPCR of ISG15, Mx1, and OAS1, mRNAs in AjKi_RML2 (E) or RoNi (F) cells treated with 10-fold serial concentrations of recombinant Chiroptera IFN-β for 24 hours. (E-F) $\Delta\Delta C_T$ values (n=3) were normalized to the average mock $\Delta\Delta C_T$ value, and the data was $\log_{10}$ transformed. Fold change of each gene at IFN-β dose was compared to mock using a two-way ANOVA with Dunnett's multiple test comparison. (A; C-F) The data is presented as the mean +/- SD. P-values for comparisons to mock cells are indicated. **** <0.0001, *** <0.001, ** < 0.01, * <0.05.

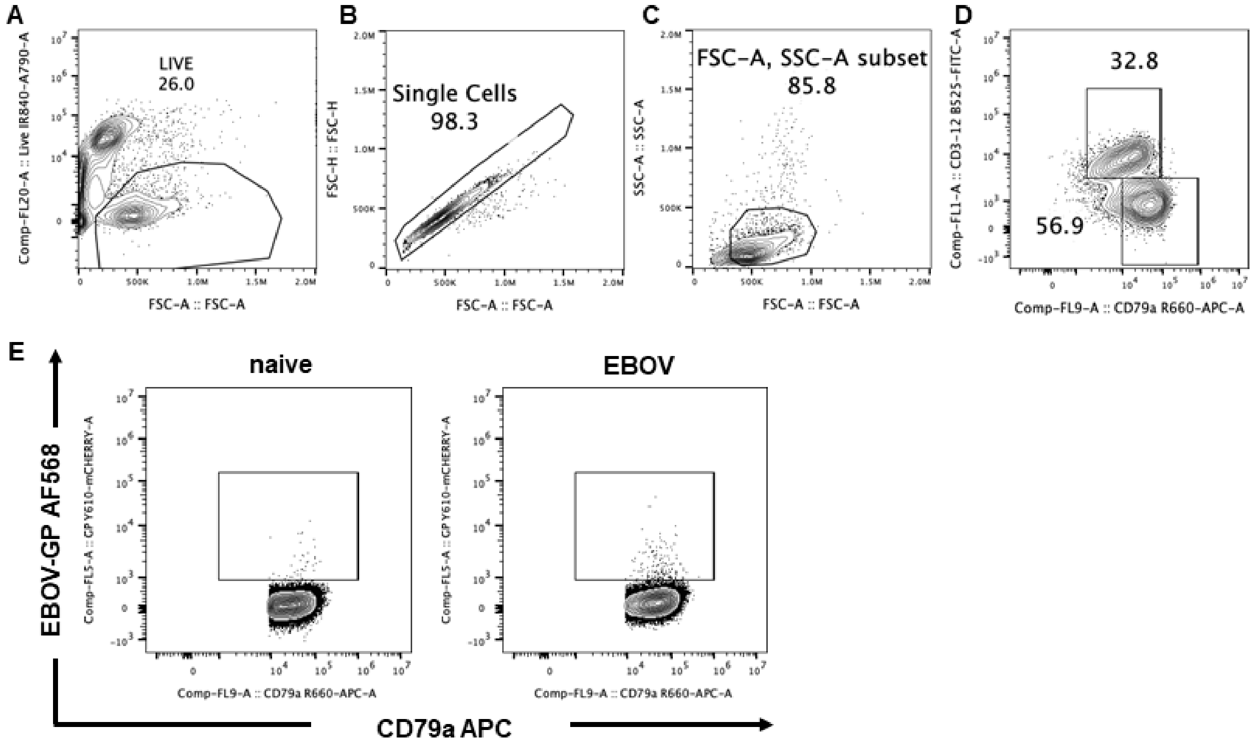

Supplementary Figure 8. Representative gating strategy for flow cytometric analysis of splenocytes

(A) Splenocytes were first gated on FSC-A vs the Live-Dead Stain to exclude dead cells. (B) Live cells were then gated to single cells using FSC-A vs FSC-H. (C) A lymphocyte gate utilizing FSC-A vs SSC-A was applied to live, single cells. (D) The live lymphocyte population was further analyzed by gating on B cells and T cells using intracellular staining of anti-CD79a APC vs anti-CD3 FITC, respectively. (E) Representative FACS contour plots showing the background staining (naïve) and staining (EBOV) of Alexa-fluor 568 conjugated recombinant EBOV GP receptor binding domain on splenic B cells
