# Supplementary material for: Jamaican fruit bats’ competence for Ebola but not Marburg virus is driven by intrinsic differences
Source: Nat Commun. 2025 Mar 25;16:2884. doi: 10.1038/s41467-025-58305-4 (PMC11937316; doi:10.1038/s41467-025-58305-4)
Supplement: Supplementary file 2 — Reporting summary [file 41467_2025_58305_MOESM2_ESM.pdf]

nature portfolio

Corresponding author(s): Dr. Vincent Munster

Last updated by author(s): Mar 12, 2025

Reporting Summary

Nature Portfolio wishes to improve the reproducibility of the work that we publish. This form provides structure for consistency and transparency in reporting. For further information on Nature Portfolio policies, see our Editorial Policies and the Editorial Policy Checklist.

Statistics

For all statistical analyses, confirm that the following items are present in the figure legend, table legend, main text, or Methods section.

n/a	Confirmed	
☐	☒	The exact sample size (*n*) for each experimental group/condition, given as a discrete number and unit of measurement
☐	☒	A statement on whether measurements were taken from distinct samples or whether the same sample was measured repeatedly
☐	☒	The statistical test(s) used AND whether they are one- or two-sided<br>*Only common tests should be described solely by name; describe more complex techniques in the Methods section.*
☒	☐	A description of all covariates tested
☐	☒	A description of any assumptions or corrections, such as tests of normality and adjustment for multiple comparisons
☐	☒	A full description of the statistical parameters including central tendency (e.g. means) or other basic estimates (e.g. regression coefficient) AND variation (e.g. standard deviation) or associated estimates of uncertainty (e.g. confidence intervals)
☐	☒	For null hypothesis testing, the test statistic (e.g. *F*, *t*, *r*) with confidence intervals, effect sizes, degrees of freedom and *P* value noted<br>*Give P values as exact values whenever suitable.*
☒	☐	For Bayesian analysis, information on the choice of priors and Markov chain Monte Carlo settings
☒	☐	For hierarchical and complex designs, identification of the appropriate level for tests and full reporting of outcomes
☒	☐	Estimates of effect sizes (e.g. Cohen's *d*, Pearson's *r*), indicating how they were calculated

Our web collection on statistics for biologists contains articles on many of the points above.

Software and code

Policy information about availability of computer code

Data collection	No software was used for data collection.
Data analysis	GraphPad Prism software, version 10.2.0;<br>Fiji for western blot quantification;<br>Immcantation version 4.3.0, BestNormalize (version 1.9.1), Rstan (version 2.32.5), and IgBLAST (version 1.18.0) for B cell receptor sequencing analysis;<br>FASTX-Toolkit, Bioconductor package DESeq2, ggplot2 (V3.4.0) as part of the tidyverse package (V1.3.2), Ingenuity Pathway Analysis, and Partek Genomics Suite for next-generation sequencing analysis;<br>FlowJo v.10.9 for flow cytometry

For manuscripts utilizing custom algorithms or software that are central to the research but not yet described in published literature, software must be made available to editors and reviewers. We strongly encourage code deposition in a community repository (e.g. GitHub). See the Nature Portfolio guidelines for submitting code & software for further information.

Data

Policy information about availability of data

All manuscripts must include a data availability statement. This statement should provide the following information, where applicable:

- Accession codes, unique identifiers, or web links for publicly available datasets
- A description of any restrictions on data availability
- For clinical datasets or third party data, please ensure that the statement adheres to our policy

The data generated in this study have been deposited in Figshare at 10.6084/m9.figshare.27854799, and the data generated in this study are provided in the Source Data file. The transcriptomics datasets have been deposited to NCBI (PRJNA1219753, [https://www.ncbi.nlm.nih.gov/sra/PRJNA1219753]). The Jamaican fruit bat genome
(GCF_021234435.1, [https://www.ncbi.nlm.nih.gov/datasets/genome/GCF_021234435.1/]) was used as the reference to analyze the transcriptomics data.

Research involving human participants, their data, or biological material

Policy information about studies with human participants or human data. See also policy information about sex, gender (identity/presentation), and sexual orientation and race, ethnicity and racism.

Reporting on sex and gender	N/A
Reporting on race, ethnicity, or other socially relevant groupings	N/A
Population characteristics	N/A
Recruitment	N/A
Ethics oversight	N/A

Note that full information on the approval of the study protocol must also be provided in the manuscript.

Field-specific reporting

Please select the one below that is the best fit for your research. If you are not sure, read the appropriate sections before making your selection.

☒ Life sciences ☐ Behavioural & social sciences ☐ Ecological, evolutionary & environmental sciences

For a reference copy of the document with all sections, see nature.com/documents/nr-reporting-summary-flat.pdf

Life sciences study design

All studies must disclose on these points even when the disclosure is negative.

Sample size	Animal study sample size was determined by minimum number of animals needed to for analysis of differences in viral replication and antibody responses. There was no pre-existing data to perform a power analysis<br>For in vitro experiments, n>/=3 replicates were used to enable statistical testing. Exact sample sizes are provided in figure legends.
Data exclusions	No data was excluded from this study.
Replication	All animal study experiments were performed one time with four bats per virus per time point which provided the necessary power to make comparisons between the Ebola and Marburg infected groups. No power calculation was used since no previous data was available to calculate expected standard deviation or expected differences between groups.<br>In vitro experiments were conducted at least one time, and where possible were repeated two or three times. The number of experiment replicates are included in the figure legends. For the experiment that was completed once in biological triplicate (Figure 6F, J and Supplementary Figure 5 F-H), the intention was to compare the difference cell lines with the same virus inoculum and this experiment generated a large number of samples and it was not feasible to repeat multiple times. No statistical analysis was applied to compare the viruses, instead we made observations on the patterns of Ebola and Marburg virus strains across the different cell lines.
Randomization	Animals were allocated at random.
Blinding	The following analysis tasks were blinded: histopathology analysis. Blinding was not applied to any other aspect of the study, because no other data was empirical and not subject to interpretation of the analyst.

Reporting for specific materials, systems and methods

We require information from authors about some types of materials, experimental systems and methods used in many studies. Here, indicate whether each material, system or method listed is relevant to your study. If you are not sure if a list item applies to your research, read the appropriate section before selecting a response.

Materials & experimental systems

n/a	Involved in the study
☐	☒ Antibodies
☐	☒ Eukaryotic cell lines
☒	☐ Palaeontology and archaeology
☐	☒ Animals and other organisms
☒	☐ Clinical data
☒	☐ Dual use research of concern
☒	☐ Plants

Methods

n/a	Involved in the study
☒	☐ ChIP-seq
☐	☒ Flow cytometry
☒	☐ MRI-based neuroimaging

Antibodies

Antibodies used

1:1000 pSTAT1 – Y701 (Cell Signaling Technology, 9167S, Lot 29)
1:1000 pSTAT2 – Y690 (Cell Signaling Technology, 88410S, Lot 5)
t1:1000 otal STAT1 (Cell Signaling Technology, 14994S, Lot 8)
1:1000 total STAT2 (Cell Signaling Technology, 72604S, Lot 6)
1:1000 RIG-I (Kerafast, 1C3, Lot 21129)
1:1000 Lamin A/C (Cell Signaling Technology, 4777S, Lot 5)
1:1000 β-tubulin (Sigma Aldrich, T8328, Lot 0000130476)
1:1000 EBOV VP24 (Sino Biological 40454-T46, Lot HD165E2825)
1:1000 EBOV VP40 (GeneTex, GTX134034, Lot 42935)
1:1000 MARV VP40 (The Native Antigen Company, MAV12450-100, Lot 21083113)
1:10,000 Donkey-anti-rabbit (GE Healthcare, NA934, Lot 17640116)
1:10,000 Sheep-anti-mouse (GE Healthcare, NA931, Lot 17638732)
1:100 Allophycocyanin-anti-CD79a (HM57, BD Biosciences 752115, Lot B422858)
1:100 Fluorescein isothiocyanate-anti-CD3e (CD3-12, BioRad MCA1477F, Lot B285646)

Validation

All of the above antibodies were validated by the manufacturer via western blot, flow cytometry, and/or immunohistochemistry
Information on validation can be found by entering the catalog number on the manufacturer's website: https://www.cellsignal.com/, https;//kerafast.com, https://www.sigmaaldrich.com, https://sinobiological.com, https://genetex.com, https://thenativeantigencompany.com, https://www.gehealthcare.com, https://www.bd.com, https://www.bio-rad.com

Eukaryotic cell lines

Policy information about cell lines and Sex and Gender in Research

Cell line source(s)

HEK293 (millipore sigma, cat # 96121229-1VL), 293T (CRL-3216) and Vero E6 (CRL-1586) cells were sourced from ATCC.
Huh-7 cells were sourced from Dr. Matsuura, Osaka University. RoNi cells were sourced from Dr. Marcel Muller.
Aji, AjKi_RML2, AjLu_RML3, AjUFi_RML6, RASKM, and PaLu1.4 cells were generated in the Munster lab and have been reported previously.

Authentication

None of the cell lines were authenticated.

Mycoplasma contamination

All cell lines were tested regularly for Mycoplasma contamination and were negative.

Commonly misidentified lines
(See ICLAC register)

No commonly misidentified cell lines were used.

Animals and other research organisms

Policy information about studies involving animals; ARRIVE guidelines recommended for reporting animal research, and Sex and Gender in Research

Laboratory animals

Jamaican fruit bats, sub-adult to adult, mixed sex

Wild animals

N/A

Reporting on sex

Although both male and female bates were used for this study, no analysis disaggregated for sex was performed due to only having 2 males and two females per group.

Field-collected samples	N/A
Ethics oversight	All animal experiments were conducted in an AAALAC International-accredited facility and were, approved by the Rocky Mountain Laboratories (RML) Animal Care and Use Committee, protocol 2022-029-E, and adhered to the guidelines put forth in the Guide for the Care and Use of Laboratory Animals 8th edition, the Animal Welfare Act, United States Department of Agriculture and the United States Public Health Service Policy on the Humane Care and Use of Laboratory Animals.

Note that full information on the approval of the study protocol must also be provided in the manuscript.

Plants

Seed stocks	N/A
Novel plant genotypes	N/A
Authentication	N/A

Flow Cytometry

Plots

Confirm that:

☒ The axis labels state the marker and fluorochrome used (e.g. CD4-FITC).

☒ The axis scales are clearly visible. Include numbers along axes only for bottom left plot of group (a 'group' is an analysis of identical markers).

☒ All plots are contour plots with outliers or pseudocolor plots.

☒ A numerical value for number of cells or percentage (with statistics) is provided.

Methodology

Sample preparation	Single cell suspension of spleen fixed in cytofix/cytoperm (BD Biosciences)
Instrument	6-laser Cytoflex LX
Software	BD FACS Diva<br>FlowJo v.10.9
Cell population abundance	N/A
Gating strategy	Live lymphocytes were gated using a FSC-A vs Live-Dead gate, followed by a FSC-A and FSC-H gate to exclude doublets and then by a standard FSC-A vs SSC-A lymphocyte gate. Then gated the proportion of CD79a APC and CD3-FITC positive cells. The number of CD79a-APC positive B cells that bound to EBOV-GP Alexa Fluor 568 was determined.

☒ Tick this box to confirm that a figure exemplifying the gating strategy is provided in the Supplementary Information.
